# Redox stress proteins are involved in adaptation response of the hyperthermoacidophilic archaeon *Sulfolobus solfataricus *to nickel challenge

**DOI:** 10.1186/1475-2859-6-25

**Published:** 2007-08-12

**Authors:** Anna M Salzano, Ferdinando Febbraio, Tiziana Farias, Giovanni P Cetrangolo, Roberto Nucci, Andrea Scaloni, Giuseppe Manco

**Affiliations:** 1 Laboratorio di Proteomica e Spettrometria di Massa, ISPAAM, Consiglio Nazionale delle Ricerche, 80147 Napoli, Italy; 2 Istituto di Biochimica delle Proteine, Consiglio Nazionale delle Ricerche, Via Pietro Castellino 111, 80131 Naples, Italy

## Abstract

**Background:**

Exposure to nickel (Ni) and its chemical derivatives has been associated with severe health effects in human. On the contrary, poor knowledge has been acquired on target physiological processes or molecular mechanisms of this metal in model organisms, including Bacteria and Archaea. In this study, we describe an analysis focused at identifying proteins involved in the recovery of the archaeon *Sulfolobus solfataricus *strain MT4 from Ni-induced stress.

**Results:**

To this purpose, *Sulfolobus solfataricus *was grown in the presence of the highest nickel sulphate concentration still allowing cells to survive; crude extracts from treated and untreated cells were compared at the proteome level by using a bi-dimensional chromatography approach. We identified several proteins specifically repressed or induced as result of Ni treatment. Observed up-regulated proteins were largely endowed with the ability to trigger recovery from oxidative and osmotic stress in other biological systems. It is noteworthy that most of the proteins induced following Ni treatment perform similar functions and a few have eukaryal homologue counterparts.

**Conclusion:**

These findings suggest a series of preferential gene expression pathways activated in adaptation response to metal challenge.

## Background

Eight of the top 100 substances on the 2005 Agency for Toxic Substances and Disease Registry priority list are toxic metals, including arsenic, chromium, cadmium, and nickel [1]. Exposure to these metals is associated with a variety of adverse health effects to humans [2-4]; however, the mechanisms leading to the development of these diseases as well as the cellular pathways modified in response to these metals exposure just begin to be understood [4].

Heavy-metal resistance mechanisms in bacteria have been shown to exist in various species [5]. Metals, such as Cd, Hg, and Ag, have a chemical preference for thiol ligands [6], deactivating enzymes that contain thiols at their active sites. In addition, metals interact with other important trace elements in cells, inhibiting their normal physiological functions [6]. Metals, such as Cu, Fe, and Mn, have direct oxidizing capacity, and other, such as Ni, Co, and Zn, cause indirect oxidative stress through uncoupling of electron transport in both respiration and photosynthesis and depletion of glutathione (GSH), leading to the accumulation of reactive oxygen species (ROS). The intracellular generation of superoxide by Cd, Ni, and Co is toxic in *Escherichia coli *[7], and superoxide dismutase is involved in protection against this metal-induced oxidative stress [7].

Investigation of the biochemical and genetic metal homeostasis and resistance mechanisms of acidophilic microorganisms, particularly of the Archaea, is virtually in its infancy. The Archaea represent challenging yet environmentally relevant systems for an increased understanding of metal resistance and homeostasis [8]. As result of the elucidation of its genome sequence in 2001 [9], hyperthermoacidophilic crenarchaeon *Sulfolobus solfataricus*, which grows between 70 and 90°C and in a pH range of 2–4 [10], is an attractive crenarchaeal model organism for functional genomic analysis. Since its isolation in the early 1980s, its preference for environments hostile to many other organisms made it an interesting source for novel thermostable enzymes. Out of the 2977 ORFs originally identified in the genome of *S. solfataricus*, 1941 genes still have no known function in TIGR's comprehensive microbial resource database [11]. Given the similarity with some pivotal eukaryal genes, the *Sulfolobus *craenarchaeon genus has been considered a simplified model for the analysis of complex topics not easily approachable in human [12]. In particular, the replication, recombination, repair, transcription and translation proteins are homologous to those of eukaryotes, despite the fact that the Archaea are prokaryotes. To fully exploit its potential, few proteomics investigations exploring gene products expressed within the cell have been reported [13-15]. However, only about 50% of the putative ORFs have been detected as really expressed in one case [14] and 10% in the second one [15]. Other proteomic studies on quantitative evaluation of protein expression changes for a defined set of genes are currently being carried out [16,17].

In this study, we investigated the adaptation response of *S. solfataricus *to challenging environments by analyzing its changes in protein repertoire. We focused on perturbations generated by chemical toxic compounds, and particularly, heavy metals on cytosolic proteins. Given the particular significance of Ni on human health, the study of Ni effects in the model system *S. solfataricus *might shed some light on similar effects in human. This manuscript reports some *Sulfolobus *proteins that significantly changed their expression levels following cell exposure to NiSO_4_.

## Results and Discussion

### Growth of *S. solfataricus *MT4 in nickel sulphate

**Figure 1:**
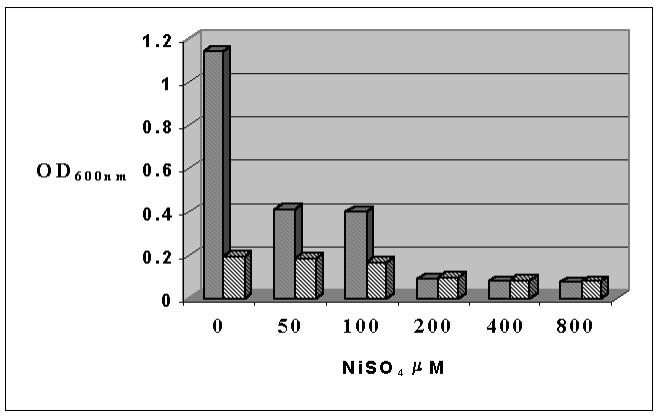
**Effect of Ni addition on the growth of *Sulfolobus solfataricus***. *Sulfolobus solfataricus *was grown aerobically in a rotary shaker, at 80°C for 19 (gray columns) or 48 h (striped columns), in the presence of 0, 50, 100, 200, 400 and 800 μM nickel sulphate. Cell reading was performed at 600 nm.

Small-scale growth experiments on *S. solfataricus *were performed to determine the highest concentration of chemical perturbing agent to be added, which maintained cells keep on growing. Six tubes were prepared each containing 3 mL of a 0.1 OD_600 nm _culture and concentrations of 0, 50, 100, 200, 400, 800 μM NiSO_4_, respectively. Tubes were incubated to 80°C, under shaking, for 19 and 48 h and then absorbance was read (Fig. 1). At concentrations of NiSO_4 _higher than 200 μM cells did not grow; therefore, a 100 μM NiSO_4 _concentration was chosen for the large-scale experiment.

Starting from the preliminary experiment mentioned above, we scaled up to 1-liter experiment for proteomic analysis. As described in the experimental section, a 2 L cell culture was split in two halves; whereas one was incubated with 100 μM NiSO_4_, at 80°C, under shaking, the other did not received metal supplement and was taken as control growing under the same experimental conditions. Cells were centrifuged and broken by sonication. Solubilized proteins from both cultures were recovered as reported in the experimental section and compared by bi-dimensional chromatography [18].

### Protein analysis

**Figure 2:**
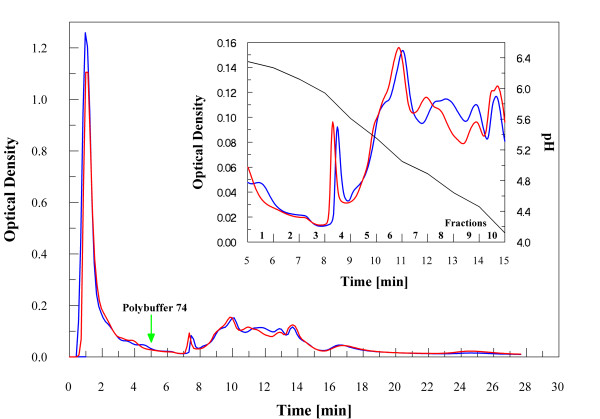
**Chromatofocusing fractionation**. Profile of the chromatofocusing in the range pH 6–4 of the protein extract (10 mg/mL) from control [blue line] and cells perturbed with 100 μM nickel sulphate [red line]. A green arrow at 5 min indicates start of elution with polybuffer 74. In the insert is shown the zoomed region of the chromatogram from 5 to 15 min corresponding to the pH elution and fractions analysed by RP-HPLC.

**Figure 3:**
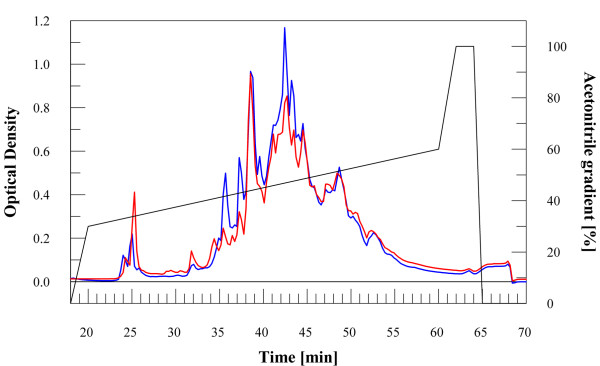
**RP-HPLC fractionation**. RP-HPLC profile of the sample n.6 from control [blue line] and cells perturbed with 100 μM nickel sulphate [red line].

**Figure 4:**
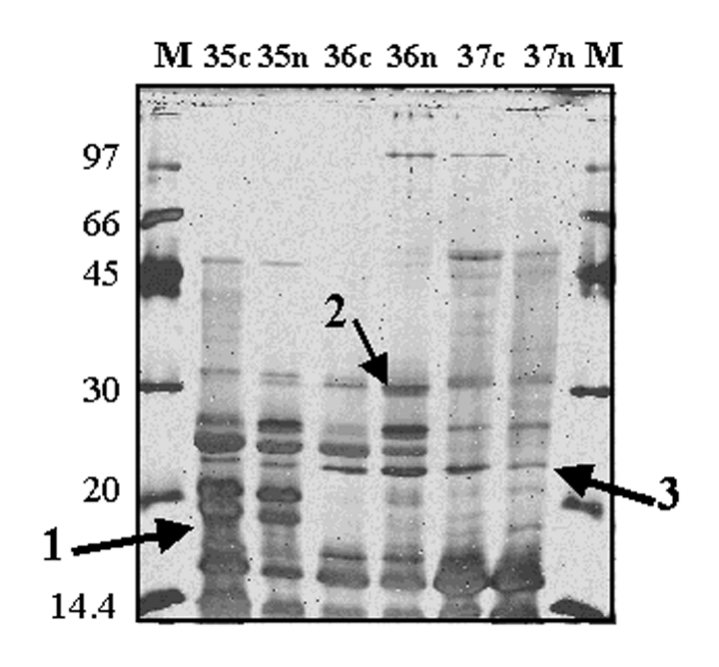
**SDS-PAGE of specific RP-HPLC fractions**. Lanes 1 and 7, protein markers; lanes 2–6, fraction 35–37 from control (c) and Ni-treated (n) samples. Fractions from RP-HPLC were desiccated and one third was loaded on the gels. Bands 1, 2 and 3 were identified as protein SSO2433, SSO2190 and SSO0886, respectively.

Samples of crude cellular extracts were separated by chromatofocusing in the range of pH 6–4, as described in the experimental section. Fig. 2 shows a typical chromatogram obtained. Fractions from four identical runs were separately pooled for both different cultures; then, each pooled fraction was fractionated by RP-HPLC, as reported in the experimental section. A total of 9 chromatographic runs were performed. Chromatographic profile of the same fractions from Ni-treated and control culture were superimposed and compared; at this stage, it was already possible to observe some differences. In Fig. 3 it is shown a typical result corresponding to reverse-phase chromatograms of chromatofocusing fractions number 6, both from control and treated cells. As shown, there was a clear difference in the chromatographic profiles in the range 30–55 min. Fractions associated to major differences in control and treated runs were concentrated and analysed by 12% SDS-PAGE. We focused on fractions showing a clear appearance/disappearance of protein bands, thus choosing only qualitative criteria of selection from electrophoresis analysis. As an example, Fig. 4 shows the comparison between the RP-HPLC fractions eluted in the 35–37 min range (Fig. 3) from treated and control culture. Bands of interest were excised and analysed by peptide mass-fingerprint analysis to identify variable proteins. Differential expression data from SDS-PAGE/peptide mass fingerprint analysis were confirmed by shotgun analysis of protein samples [19].

By using this methodology, we identified different proteins significantly varying their abundance in the treated versus the control sample (manuscript in preparation). Table 1 shows selected proteins detected only in the control or in the Ni-treated samples. Five proteins were detected only in the non-treated sample, whereas eleven proteins were detected only in the nickel-treated sample. Proteins disappearing after Ni treatment were a NAD-dependent malic enzyme involved in energy uptake, a group of four hypothetical proteins of unknown function made up of three CO dehydrogenase subunits (large chain cutA-6 and small chain cutC-1) and acetyl-coenzyme A synthetase. Carbon monoxide dehydrogenase/acetyl-CoA synthase (CODH/ACS) complex in *M. thermophila *is part of a five-subunit complex consisting of α, β, γ, δ, and ε subunits [20]. The multienzyme complex catalyzes the reversible oxidation of CO to CO_2_, transfer of the methyl group of acetyl-CoA to tetrahydromethanopterin (H_4_MPT), and acetyl-CoA synthesis from CO, CoA, and methyl-H_4_MPT. The α and ε subunits are required for CO oxidation. The γ and δ subunits constitute a corrinoid iron-sulfur protein that is involved in the trans-methylation reaction. Isolated β subunit contains significant amounts of nickel. The β subunit harbors cluster A, a NiFeS cluster, which is the active site of acetyl-CoA cleavage and assembly. Although the β subunit is necessary, it is not sufficient for acetyl-CoA synthesis; interactions between the CODH and the ACS subunits are required for cleavage or synthesis of the C-C bond of acetyl-CoA. These interactions include intra-molecular electron transfer reactions between the CODH and ACS subunits [21]. The role of this complex in the adaptation to metal stress conditions is currently unknown.

**Table 1:** Down/up-regulated proteins detected following challenge of *S. solfataricus *with 100 μM NiSO_4_.

**Down-regulated proteins**	
NAD-dependent malic enzyme (malate oxidoreductase)	**SSO2869**
Hypothetical protein	**SSO1388**
Carbon monoxide dehydrogenase. large chain (cutA-6)	**SSO2942**
Carbon monoxide dehydrogenase, small chain (cutC-1)	**SSO2433**
Acetyl-coenzyme A synthetase	**SSO1314**

**Up-regulated proteins**	

Putative peroxiredoxin	**SSO2255**
Hypothetical protein	**SSO2253**
Pyrodoxyn *ethylene *inducible	**SSO0570**
Hypothetical protein	**SSO1152**
Transcription regulator (exsB) related protein	**SSO0016**
Maltooligosyltrehalose synthase (treY)	**SSO2095**
NAD-specific glutamate dehydrogenase (gdhA-1)	**SSO1457**
NAD-specific glutamate dehydrogenase (gdhA-2)	**SSO1907**
Carbon monoxide dehydrogenase large chain (cutA-1)	**SSO1209**
Hypothetical protein	**SSO0886**
Hypothetical protein	**SSO2190**

**Figure 5:**
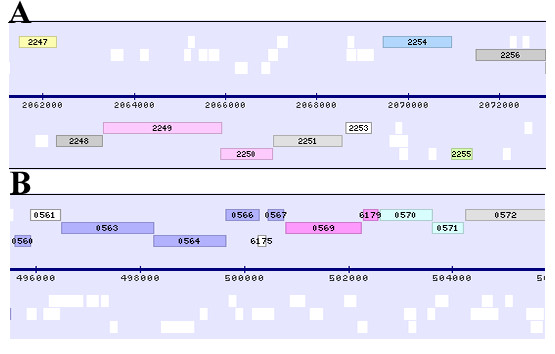
**Genome location of selected identified ORFs**. **Panel A. **Genome location of ORF SSO2255 (green) and SSO2253 (white) in front of a cluster of genes suggested to be involved in DNA repair/recombination (Rad32/Mre11: SSO2250; Rad50: SSO2249; NurA: SSO2248; MlaA/HerA: SSO2251). **Panel B. **Genome location of ORFs SSO0570 and SSO0571 (green). Pictures were adapted from ref. [68].

On the contrary, several proteins of particular interest occurred in the group of up-regulated proteins (Table 1). In particular, the first one is the protein Bcp3 corresponding to *S. solfataricus *ORF SSO2255, which is annotated as putative peroxiredoxin (Prx). Four paralogous ORFs, with similarity to Prxs and annotated as Bcp1-4 (SSO2071, SSO2121, SSO2255 and SSO2613, respectively), are present in the *S. solfataricus *genome [9]. *S. solfataricus *Bcp bearing the greatest similarity to other Prxs in the GenBank Database is Bcp2, which encodes for a protein of 215 amino acids. Bcp2 reveals 40% of identity with 1-Cys Human PRDX6. Analysis in *S. solfataricus *of this gene and the protein encoded by it has been recently reported [22]. Its role in oxidative stress was investigated by transcriptional analysis of RNA isolated from cultures stressed by various oxidative agents. Its specific involvement was confirmed by a considerable increase of the Bcp2 transcript following induction by H_2_O_2_. Using dithiothreitol as an electron donor, this enzyme acts as a catalyst in H_2_O_2 _reduction and protects plasmid DNA from nicking by the metal-catalysed oxidation system. Western blot analysis revealed that the Bcp2 expression was induced as a cellular adaptation in response to the addition of exogenous stressors. The results obtained indicate that Bcp2 plays an important role in the peroxide-scavenging system in *S. solfataricus *[22] and it is expected that the other genes have a similar role. No biochemical data are available on the other three bacterial Prx forms. In *P. horikoshi *it has been reported that Bcp2 protein is induced as well following oxidative stress [23]. The observation that Bcp3 appears after nickel treatment could be interpreted as due to a similar specific induction, thus supporting the idea that is involved in the recovery from metal-induced stressing conditions. The genome location of the corresponding gene is shown in Fig. 5A.

A second protein worth of noting is the hypothetical ORF SSO2253 (Table 1), which is located at the same locus and on the same (lagging) strand of Bcp3, but head of a cluster of four genes (see Fig. 5A) containing Rad30/Mre11 and Rad50 homologues involved in DNA recombination and repair in Eukarya [24]. This operon in Archaea contains two additional conserved ORFs. The first one has recently been reported to code for a nuclease, suggesting that the operon is linked to double strand break repair and recombination in Archaea [25]. The second one codes for a novel 60-kDa protein (termed MlaA/HerA) with a bimodal helicase activity [26,27]. MlaA/HerA is fused with Mre11 in *M. thermoautotrophicus*, suggesting that MlaA is functionally linked to the Mre11 complex [27]. The conservation of this archaeal operon suggests that these four proteins could participate in the same repair/recombination pathway, but no evidences have been provided of such a role *in vivo*. Till now no clues are available for a role of SSO2253. The gene is conserved at the same position also in *S. acidocaldarius *and *S. tokodai*, whereas is on the opposite strand in *P. torridus*. The stop codon for SSO2253 is at 87 nt from the ATG starting codon of SSO2251 (MlaA/HerA). A sequence analysis with the program PredictProtein (see Methods), allowed predicting for this protein a ferritin-like fold and PSI-Blast predicted weak similarity to some archaeal and bacterial rubrerytrins. However, no similarity was detected with a second *Sulfolobus *rubrerytrin, namely SSO2642. Accordingly, our results suggest the possibility of a coordinate action of all the proteins at this locus in oxidative stress recovery.

Induced expression of putative "ethylene-inducible" gene SSO0570 was also observed; its protein product belongs to the Sor/SNZ family, involved in the biosynthesis of vitamin B6 [28]. Pyridoxine, pyridoxal, and pyridoxamine are collectively called vitamin B6. Being a cofactor for many enzymatic reactions, especially those involved in amino acid metabolism, vitamin B6 is required for all organisms [29]. Two distinctive *de novo *pyridoxine biosynthesis pathways have been identified. One well-characterized pathway exists in some eubacteria such as *Escherichia coli*. In this pathway, pyridoxine is synthesized from 1-deoxy-D-xylulose-5-phosphate and 4-phosphohydroxy-L-threonine through pyridoxine synthase proteins Pdx2A and Pdx2J [30,31]. The second pathway has just begun to be elucidated in fungi, archaeabacteria, and some eubacteria. In these organisms, pyridoxine may be synthesized from glutamine and ribose 5-phosphate or ribulose 5-phosphate through two families of conserved proteins: the singlet oxygen resistance (SOR)/snooze (SNZ) family and the SNZ proximal open reading frame (SNO) [32]. A homologous gene found in *Cercospora *fungi is SOR1, a gene with demonstrated activity against singlet oxygen [33]. Preponderance of work on reactive oxygen species has focused on reduced forms of oxygen, hydrogen peroxide (H_2_O_2_), radicals superoxide (O_2_^•2^) and the hydroxyl radical (OH•); contrarily, singlet oxygen (^1^O_2_), a highly reactive but non-radical species of active oxygen, has received relatively less attention. ^1^O_2 _is primarily generated via photosensitization [34]. Photosensitizers and photoactizers are a diverse and ubiquitous group of compounds and natural products synthesized by plants, microorganisms, and protozoans. Singlet oxygen reacts rapidly with biomolecules such as lipids, proteins, and nucleic acids and shows almost universal toxicity to cells. Cercospora fungi use production of cercosporin, a photosenthitizer, to parasitize plants [35]. Despite the prevalence of photosensitizers, little is known about effective cellular defences against them and the ^1^O_2 _they generate upon illumination. Defenses effective against radical and reduced oxygen species (e.g., superoxide dismutase, catalase, peroxidases) are not effective against ^1^O_2_. Vitamin B6 has been demonstrated to be able to quench superoxide and singlet oxygen [36,37]. The discovery of the involvement of the biosynthetic route to vitamin B6 in the archaeon *S. solfataricus *as mean for recovery from Ni-induced oxidative stress could *i*) suggest production of ^1^O_2 _under such conditions, ii) confirm its role as bioscavenger for ^1^O_2 _and O_2_^•2 ^[36,37] and iii) suggest this mechanism as very ancient, being *S. solfataricus *a craenarcheon. However, although there are several papers showing that nickel alone or in combination with other compounds induces ROS formation (comprising singlet oxygen production) and a few supporting the notion of singlet oxygen production in cells [38,39], Ni transition metal-chelates are good singlet oxygen quenchers. The point is if nickel induces *per se *singlet oxygen or if this is a secondary effect. The possibility of a secondary effect could not be discharged. Singlet oxygen for example can be produced by a dismutation reaction from superoxide ions or a reaction between hydrogen peroxide and superoxide ions in acidic environment.

Quite interestingly, in the genome location (Fig. 5B) nearby the gene is present ORF SSO0571 coding for a predicted glutamine amidotransferase involved in pyrodoxyn biosynthesis, which has been found co-eluting with "ethylene-inducible protein" SSO0570 in the Ni-treated sample (data not shown). In conclusion, ORFs SSO0570 and SSO0571 represent the archaeal synthase/glutaminase bifunctional enzyme involved in *de novo *vitamin B6 biosynthesis. The occurrence of both subunits in the same chromatofocusing fraction may suggest a possible physical association; actually, physical interaction between SNZ1 and SNO1 has been already reported in yeast [40].

Other two proteins, namely ORF SSO0866 and ORF SSO2190, were found induced following Ni stress. These proteins are annotated as hypothetical proteins because only a very weak similarity to a molecular chaperon (COG0443) and a dithiol-disulfide isomerase involved in polyketide biosynthesis (COG2761), respectively, was detected with a BLAST search. Their involvement in response to redox stress could be only hypothesized, as already observed in other organisms for this class of enzymes. These hypothetical proteins were found in a locus rich in proteins of unknown function.

The protein SSO1152 has similarity with the peptidase U62 of the PmbA/TldD family. This protein, which has been originally described as a gyrase modulator in *E. coli *[41], is a Zn-dependent peptidase [42]. The finding of this protein only in the Ni-treated sample is particularly significant in view of the observation that the unique gyrase reported from *S. solfataricus*, namely reverse gyrase, was detected in both samples subjected to shotgun sequencing (data not shown). This could suggest a physical association between the putative peptidase U62 and reverse gyrase in only the Ni-treated sample. Recently, it has been reported that reverse gyrase activity and immuno-related material decreases in *Sulfolobus *cells treated by MMS [43]; this decrease has been attributed to a metal-dependent protease. The speculation that the putative peptidase U62 might bind reverse gyrase and fulfill this role could be a work hypothesis for further experimentation.

The protein SSO0016 encodes for a transcription regulator (*exsB*) related protein. This family includes putative transcriptional regulators from Bacteria and Archaea. In *R. meliloti*, a species in which the *exo *genes make succinoglycan, a symbiotically important exopolysaccharide, *exsB *is located nearby and affects succinoglycan levels [44]. In *A. viscosus*, the homologous gene is designated ALU1 and is associated with an aluminium tolerance phenotype. The function is unknown [45]. In *Sulfolobus *SSO0016 is adjacent to a putative permease (SSO0015) and to a putative membrane protein (SSO0014), both of unknown function.

Threalose is a well-known sugar, which serves as carbon source but is also involved in adaptation to physical and chemical stresses in several organisms. In *Anabaena *7120 cells, it has been reported induction of enzyme maltooligosyltrehalose synthase (MTSase) following osmotic stress [46]. Santos group [47] reported that the level of OtsA (a gene involved in threalose synthesis) was enhanced (approximately twofold) by osmotic, oxidative and acid stress, whereas the level of TreS (threalose degradation) remained constant, or decreased, under identical stress conditions. Therefore, the OtsA-OtsB pathway plays an important role in the synthesis of trehalose in response to stress. Furthermore, in the papers of Wright and Dratz, cited above [14,15], the protein TreY was identified in both cases, although growth conditions were the same as reported by us. The up-regulation of this enzyme in the *Sulfolobus *Ni-treated sample is in agreement with the above report. The structure reported from *Deinococcus radiodurans *suggests the presence of metals at the active site [48].

The two NAD-specific glutamate dehydrogenases have also been found in Ni-treated cells. The increase/induction of these enzymes could be related to recovery from oxidative stress, as observed for the homologous enzyme from plants [49], but the exact pathway of how this happens in Archaea remains to be determined.

Nickel ion is specifically incorporated into Ni-dependent enzymes, often via complex assembly processes requiring accessory proteins and additional non-protein components, in some cases accompanied by nucleotide triphosphate hydrolysis. To date, nine Ni-containing enzymes are known: urease, NiFe-hydrogenase, carbon monoxide dehydrogenase, acetyl-CoA decarbonylase/synthase, methyl coenzyme M reductase, certain superoxide dismutases, some glyoxylases, aci-reductone dioxygenase, and methylenediurease. Seven of these enzymes have been structurally characterized, revealing distinct metallocenter environments in each case [50]. Among these enzymes, three kinds of CO dehydrogenases were found in this analysis. In particular, SSO2942 and SSO2433 appeared only in the control; SSO1209 which was detected only in the Ni-treated cells (Table 1). All the proteins are annotated as homologs of Cox/Cut proteins [51], but their genome location in *S. solfataricus *is not the same. Since in some cases, specific CO dehydrogenases have been implicated in oxidative stress response [52], the selective presence of these subunits in *S. solfataricus *control and treated samples could be related to a switch from energy up-take to oxidative stress recovery.

## Conclusion

In this study, we described a proteomics investigation based on bi-dimensional chromatography focused to understand the mechanisms that underlay the recovery from Ni-induced stress in *S. solfataricus*. We identified several proteins that specifically were associated with the Ni-treated sample, providing evidences on its specific induction. Most of these proteins are largely endowed with the ability to trigger recovery from oxidative and osmotic stress in other systems. However, our findings suggest that other preferential gene expression pathways are activated in adaptation response to metal challenge. Apart from proteins with an on/off response discussed here, there is a lot that just change their expression level (data not shown). Currently, we are involved in the quantitative evaluation of these proteins to define a comprehensive picture of nucleotide sites in the promoter region of genes whose expression varied as result of Ni-sensitive regulators action. These results will provide a better comprehension of biochemical processes related to metal stress resistance in *S. solfataricus*, allowing defining the molecular bases of adaptation response.

## Methods

### Materials

Gelrite gellan gum, D-glucose, trypsin, α-cyano-4-hydroxycinnamic acid, Coomassie Brilliant Blue R-250 and salts were from Sigma. Yeast extract and casamino acids were obtained from Difco Laboratories. Polybuffer 74 was purchased from Pharmacia; molecular mass protein markers were from BioRad. All other reagents and HPLC-grade solvents were from Fluka.

### Growth of the archaeon *Sulfolobus solfataricus *strain MT4

Fresh *S. solfataricus *MT4 cells were prepared starting from cell stabs stored at -80°C, which were streaked on plates containing 0.8% Gelrite gellan gum medium supplemented by 0.1% D-glucose and incubated at 80°C, for 3 days. A pre-culture was prepared by incubating 70 μL of spots with 100 mL of medium, pH 3.7–3.8, containing 1 g/L yeast extract, 1 g/L casamino acids, 2.5 g/L (NH_4_)_2_SO_4_, 3.1 g/L KH_2_PO_4_, 20 mg/L MgSO_4_·7H_2_O, 25 mg/L CaCl_2_·2H_2_O, 1.8 mg/L MnCl_2_·4H_2_O, 4.5 mg/L Na_2_B_4_O_7_·10H_2_O, 0.22 mg/L ZnSO_4_·7H_2_O, 0.05 mg/L CuCl_2_·2H_2_O, 0.03 mg/L Na_2_MoO_4_·2H_2_O, 0.03 mg/L VaSO_4_·2H_2_O, 0.01 mg/L CoSO_4_·7H_2_O and 0.1% D-glucose. Cells were grown aerobically in a rotary shaker, at 80°C, for 19 or 48 h; growth was monitored by measuring the turbidity at 600 nm. On the other hand, a 2 L cell culture was grown under the same conditions and split in two halves at starting OD_600 nm _= 0.1; whereas one received 100 μM NiSO_4 _(final concentration), the other was taken as control. Cultures were incubated at 80°C under shaking for 19 h. Cells were recovered by centrifugation at 6000 *g*, for 10 min. About 1.5 g of cells were obtained for each culture, which were re-dissolved in 3.5 vol of 20 mM Tris-HCl buffer pH 8, containing 1 mM PMSF, 0.5 mM EDTA, 5 mM DTT. Cells were broken by sonication (Braun). The cellular debris was removed by centrifugation at 20200 *g*, for 15 min, and soluble proteins were recovered. Protein concentration of the supernatant was determined using the Bradford Protein Assay (Sigma). Supernatants were stored at -80°C until used. Proteomic experiments were performed in triplicate using each time protein extracts from three different bacterial cultures.

### Bi-dimensional chromatography

Aliquots of protein extracts (10 mg/mL) from *S. solfataricus *cells grown under standard conditions and in Ni-supplemented medium were analysed by chromatofocusing on a FPLC AKTA apparatus (Pharmacia), using a column Mono P HR 5/5 (Pharmacia) equilibrated in 25 mM Bis-Tris buffer, pH 6.3. Proteins were eluted in the pH ranges 6–4 by using 1:10 v/v diluted Polybuffer 74 pH 4.0 (Pharmacia), at flow rate of 1 mL/min. Nine eluted fractions (3 ml each) were collected for control and Ni-perturbed cell extracts. After appropriate volume reduction, fractions were analysed by RP-HPLC on a Dionex P580 apparatus, equipped with a PDA-100 photodiode Array detector, using a Chrompack Vydac C4 column (250 × 4.6 mm), equilibrated in 0.1% TFA. Proteins were eluted in 70 min using non-linear gradient of 0–100% acetonitrile in 0.08% TFA, at a flow rate of 1 mL/min.

### Electrophoresis

One-dimensional SDS-polyacrylamide gel electrophoresis was performed on the BioRad Mini-Protean II system (7 × 10 cm) as described by Laemmli [53]. Fractions from RP-HPLC were evaporated with a Savant and re-suspended in 30 μl of loading buffer [54]. Ten μl were loaded onto 12% acrylamide gels (1.5 mm thick), which were stained with 0.2 % Coomassie Brillant Blue G250 or silver nitrate [55].

### Mass spectrometry

Bands from SDS-PAGE were excised from the gel, S-alkylated and digested with trypsin as previously reported [56]. Peptide digests were desalted using μZipTipC18 pipette tips (Millipore, USA) and loaded on the MALDI target together with CHCA as matrix, using the dried droplet technique. Samples were analysed with a Voyager-DE PRO spectrometer (Applera, USA). Mass spectra for PMF experiments were acquired in reflectron mode; internal mass calibration was performed with peptides from trypsin autoproteolysis. Spectra were elaborated using the DataExplorer 5.1 software (Applera, USA) and Mascot software (Matrix Science, UK) [57] was used to identify spots from a NCBI non-redundant database. Candidates with program scores > 79 were further evaluated by the comparison with Mr experimental values obtained from SDS-PAGE. Eventual occurrence of protein mixtures was ascertained by sequential searches for additional protein components using unmatched peptide masses.

RP-HPLC fractions showing differences in SDS-PAGE were also analyzed by a shotgun proteomic approach. In this case, fractions before and after fraction of interest were also analyzed in comparison. Protein mixtures were digested with 5 ng of trypsin in 50 mM ammonium bicarbonate, pH 8. Resulting peptide mixtures were analysed by μLC-ESI-IT-MS/MS using a LCQ Deca Xp Plus mass spectrometer (ThermoFinnigan, USA) equipped with an electrospray source connected to a Phoenix 40 pump (ThermoFinnigan, USA). Peptide mixtures were separated on a capillary ThermoHypersil-Keystone Aquasil C18 Kappa column (100 × 0.32 mm, 5 μm) (Hemel Hempstead, UK) using a linear gradient from 10% to 60% of acetonitrile in 0.1% formic acid, over 60 min, at flow rate of 5 μl/min. Spectra were acquired in the range *m/z *200–2000. Three injections were analyzed for each sample. Acquisition was controlled by a data-dependent product ion scanning procedure over the three most abundant ions, enabling dynamic exclusion (repeat count 2 and exclusion duration 3 min). The mass isolation window and collision energy were set to *m/z *3 and 35%, respectively. Data were elaborated using the BioWorks 3.1 software provided by the manufacturer. Sequest algorithm [58] was used to identify proteins μLC-ESI-IT-MS/MS experiments. Proteins were identified by comparison of tryptic peptide product ion mass spectra against those generated from a database [59] containing the traduced *S. solfataricus *P2 sequence [9] from, together with trypsin and keratins. Strain MT4 has been reported very similar to strain P2 based on criteria of genomic homology, physiological properties [60-62] and differences in specific genes [63,64]. Sequest parameters included selection of trypsin with up to 2 missed cleavage sites and dynamic mass modification associated to Met oxidation. Identified proteins were ranked in ascending order according to consensus scores and false positive identifications minimized by filtration against 4 of the following criteria: Xcorr > 2, ΔCn > 0.2, Sp > 400, rsp < 5, ions > 30% [65]. Where appropriate, protein identifications were checked manually to provide for a false positive rate of < 1% using Xcorr and ΔCn values described and validated elsewhere [66]. In the case of 1-DE bands, identified proteins were further evaluated by the comparison with Mr experimental values. In both cases, reproducibility and reliability of the results was subjected to the following criteria: i) proteins with more than 2 peptides were considered as reliable proteins, ii) single peptide protein identifications were considered reliable if they were found repeated in the same fraction number of other injections [19]. Differently expressed components were identified as proteins that were always present/absent in a specific RP-HPLC fraction obtained from cells grown in a certain medium, which were always found as absent/present in the corresponding RP-HPLC fraction obtained from cells grown under different experimental conditions.

### Sequences analysis

Sequences were analysed with program PredictProtein [67] or tools available under Sulfolobus solfataricus P2 web site [68].

## Competing interests

The author(s) declare that they have no competing interests.

## Authors' contributions

AMS performed mass spectrometry experiments. FF participated in the design of the study and helped in bi-dimensional chromatography and analysis of results. TF carried out bi-dimensional chromatography and bibliographic research. GC participated in bi-dimensional chromatography. RN participated in the design of the study. AS directed the mass spectrometry experiments, helped in writing the paper. GM Conceived the study, analysed and interpreted the results, drafted the paper. All Authors read and approved the final manuscript.
